# Chitosan Is Necessary for the Structure of the Cell Wall, and Full Virulence of *Ustilago maydis*

**DOI:** 10.3390/jof8080813

**Published:** 2022-08-02

**Authors:** José Alejandro Sánchez-Arreguin, M. Lucila Ortiz-Castellanos, Angélica Mariana Robledo-Briones, Claudia Geraldine León-Ramírez, Domingo Martínez-Soto, José Ruiz-Herrera

**Affiliations:** 1 Centro de Investigación y de Estudios Avanzados del Instituto Politécnico Nacional, Departamento de Ingeniería Genética, Unidad Irapuato, Km 9.6, Libramiento Norte, Carretera Irapuato-León, Irapuato 36821, Guanajuato, Mexico; alejandro.sanchez@cinvestav.mx (J.A.S.-A.); lucila.ortiz@cinvestav.mx (M.L.O.-C.); angelica3robledo@gmail.com (A.M.R.-B.); claudia.leon@cinvestav.mx (C.G.L.-R.); 2 Tecnológico Nacional de México, Instituto Tecnológico Superior de Los Reyes, Los Reyes 60300, Mich, Mexico; dmartinezsot@umass.edu; 3 Department of Biochemistry and Molecular Biology, University of Massachusetts Amherst, Amherst, MA 01003, USA

**Keywords:** *Ustilago maydis*, chitosan, cell wall, filamentation, virulence

## Abstract

Smut fungi comprise a large group of biotrophic phytopathogens infecting important crops, such as wheat and corn. *U. maydis* is a plant pathogenic fungus responsible for common smut in maize and teocintle. Through our analysis of the transcriptome of the yeast-to-mycelium dimorphic transition at acid pH, we determined the number of genes encoding chitin deacetylases of the fungus, and observed that the gene encoding one of them (UMAG_11922; *CDA1*) was the only one up-regulated. The mutation of this gene and the analysis of the mutants revealed that they contained reduced amounts of chitosan, were severely affected in their virulence, and showed aberrant mycelial morphology when grown at acid pH. When the *CDA1* gene was reinserted into the mutants by the use of an autonomous replication plasmid, virulence and chitosan levels were recovered in the retro mutant strains, indicating that the *CDA1* gene was involved in these features. These data revealed that chitosan plays a crucial role in the structure and morphogenesis of the cell wall during mycelial development of the fungus, and that in its absence, the cell wall becomes altered and is unable to support the stress imposed by the defense mechanism mounted on by the plant host during the infection process.

## 1. Introduction

*Ustilago maydis* is a biotrophic fungal plant pathogen that causes common smut in maize and teocintle [1,2]. In infected maize leaves, *U. maydis* induces the formation of characteristic tumors, chlorosis, and synthesis of anthocyanins. Usually, when plants are infected in the greenhouse, sori develop in the male or female flower tissue, in which florets become substituted by teliospores [3,4,5,6]. It has been shown that *U. maydis* pathogenesis is dependent upon changes in its morphology to generate specific cell types capable of host penetration, host colonization, and its dispersal. The infection of maize by *U. maydis* requires that haploid, budding yeast cells of compatible mating types fuse and grow as dikaryotic filamentous cells, which penetrate and colonize the plant tissue, giving rise to specific tumors. Within these tumors, the dikaryon proliferates and changes its morphology into melanized spores that eventually break the plant cells and tumors to disperse. Eventually, the spores germinate to produce haploid progeny capable of reinitiating the fungal life cycle [7,8,9,10,11].

It is important to stress that during the full cell cycle, the wall is an essential organelle that provides the structure and integrity to the cell. An indispensable component of this cell wall that contributes to its strength and integrity is chitin [12]. Chitin is a linear polymer of β-(1-4)-linked *N*-acetylglucosamine (GlcNac) units synthesized by chitin synthases. In a number of fungi, chitin is partially deacetylated to produce chitosan, a derivative that contains variable proportions of β-1-4-glucosamine. This process of enzymatic deacetylation is carried out by chitin deacetylases (CDA; EC 3.5.1.41). The occurrence of this process is responsible for the phenomenon of the cell walls of diverse fungi containing both chitin and chitosan, whose quantity and proportions depend on different factors, including the growth phase [6,13,14,15,16]. Despite containing eight chitin synthase genes (*CHS*) and three synthase regulatory genes (*CSR*) [17,18], *U. maydis* chitin synthase isoforms, Chs6 and Csr2, produce the majority of vegetative cell wall chitin, part of which is further on converted to chitosan. This process is carried out by eight chitin deacetylases, Classes 1 to 8, that are bound to the cell wall through GPI (glycosylphospatidylinositol) bonds [19]. This type of association is the way in which Cdas of most fungi are anchored to the cell wall, but not all use this mechanism; in other fungi, Cdas are secreted [20,21,22,23]. In this way, in plant pathogenic fungi, chitin deacetylase activity may block host recognition and the degradation of their cell walls by plant chitinases [24]. Thus, evidence has shown that it is chitin that carries out the first interaction between plant pathogenic fungi and their hosts, inducing an immune response of the plant host. Accordingly, it has been observed that chitin deacetylation by deacetylases with the formation of chitosan protects the pathogen from the action of chitinases that possess a NodB motif [25,26,27].

In some model fungi, different effects have been observed when any of the genes that synthesize chitin deacetylases are absent. In *Magnaporte oryzae*, it was observed that the mutation of three of the eight genes encoding chitin deacetylases did not affect virulence [28,29]. On the other hand, strains of *Cryptococcus neoformans*, where *CHS3, CSR2,* or all three chitin-deacetylase-encoding genes are mutated, were deficient in chitosan production and displayed a common set of phenotypes, including sensitivity to a variety of cell wall inhibitors. This indicates that chitosan is essential for the proper maintenance of cell wall integrity in this fungus [18,30]. Nevertheless, in some systems, it has been reported that certain Chs are quantitatively or qualitatively more important than others [17]. Thus, Chs3 from *Saccharomyces cerevisiae* is responsible for the synthesis of the chitin ring during bud emergence, for chitosan formation in the spore wall, and for the synthesis of most of the wall chitin of the yeast in the function of chitin synthases 2 and 3 in the *S. cerevisiae* cell cycle [31].

Interestingly, we found that one of the *U. maydis* chitin deacetylases (*CDA1*) was induced more than 10 times when *U. maydis* infected *Arabidopsis thaliana* [32]; also, the same gene was found up-regulated during *U. maydis* dimorphism induced by growth at an acid pH [33].

With this background, we proceeded to characterize *CDA1* and determine the biological role of chitosan during the vegetative growth and pathogenic stages in *U. maydis*. Analysis of the deleted strains demonstrated that *CDA1* is important for maintaining wall integrity during cell growth, and that *CDA1* absence severely impaired the virulence of this Basidiomycota fungus.

## 2. Materials and Methods

### 2.1. Fungal Strains, Culture Media, and Growth Conditions

The *U. maydis* wild type strains, FB1 (*a1b1*) and FB2 (*a2b2*) [1], as well as the *a1b1*∆*cda1* (*a1b1 cda1*::hyg) and *a2b2*∆cda1 (*a2b2 cda1*::hyg) mutants isolated in this study, were used in the present work. All the strains were maintained in 50% glycerol at −70 °C, and recovered in liquid complete medium (CM) according to Holliday (1974). For mutant isolation, YEPS growth medium (1% yeast extract, 2% sucrose, and 2% peptone) was used. For phenotypic analyses, cells were incubated in MM minimal liquid medium [34], normally of pH 7 (MM pH 7) unless otherwise indicated, under shaking conditions (125–150 rpm) at 28 °C for 18 h. Growth was measured by their absorbance at 600 nm. Agar plates of MM pH 7 with or without additions were used for growth on a solid medium, except when otherwise indicated.

### 2.2. Techniques for Nucleic Acids Manipulation

DNA from *U. maydis* was measured under the conditions described by Hoffman and Winston [35]. To isolate DNA, cells were incubated in a complete medium, plus the selective antibiotic when necessary, under shaking conditions (125–150 rpm) at 28 °C for 18 h, and recovered by centrifugation. DNA and RNA concentrations and quality were determined with a Nanodrop 2000 Spectrophotometer (Thermo Scientific, Waltham, MA, USA), and their integrity was confirmed by gel electrophoresis. Total RNA was isolated using the Direct-zol™ RNA MiniPrep Plus Kit (Zymo Research, Orange, CA, USA).

### 2.3. Polymerase Chain Reaction (PCR) Conditions

PCR reactions were carried out with the Platinum *Taq* DNA polymerase (Invitrogen, Carlsbad, CA, USA), using the following program: initial cycle of 94 °C for 5 min, amplification (30–35 cycles) at 94 °C for 30 s, followed by annealing at primer specific temperature for 60 s, and polymerization at 72 °C (1 min per kb of DNA target length). The PCR system (Boehringer, Mannheim, Germany) and the PCR products (Invitrogen) were used following the manufacturer’s instructions.

### 2.4. Isolation of Ustilago Maydis Mutants

*U. maydis* ∆*cda1* mutants of FB1 (*a1b1*) and FB2 (*a2b2*) strains were obtained by the double joint PCR method [36]. The primers used to construct the deletion cassettes of the *CDA1* gene are shown in Supplementary Table S1. PCR reactions were conducted as described above. For the first PCR reaction, 100 ng of genomic DNA template was used for amplification of the 5′ and 3′ flanking regions of the *CDA*1 gene (primers, CDS1-PF1 and CSD2-PQR2, CDS1-UQF3, and CSD1-UTR4). In a parallel PCR, 5 ng of pHyg101 providing hygromycin resistance [37] was used for amplification of the HPH marker. In the third PCR, deletion cassettes were amplified using 1 μL of the purified second PCR products as a template, and the CDS1-NF5 and CDS1-NR6 primers 

Once the correct fragment size and restriction pattern were confirmed, the DNA was purified using Pure Link Quick PCR purification columns (Invitrogen, Carlsbad, CA, USA), and 5 μg of purified DNA was used for PEG-mediated protoplast transformation of the wild type strains. Briefly, protoplasts of *U. maydis* were obtained with a pre-treatment, as described by Elorza et al. [38], followed by treatment with the *Trichoderma harzianum* lysing enzymes (Sigma-Aldrich, St. Louis, MO, USA). Transformation was under hypertonic conditions with the corresponding disruption cassette, and the transformed cells were inoculated on thin plates of YEPS light solid medium for 24 h, covered with a layer of the same medium containing hygromycin 350 μg/mL, and further on incubated for one week at 28 °C [39]. The verification of the generated mutants was carried out by PCR (Supplementary Figure S1).

### 2.5. Chitosan Measurement

Quantification of the chitosan in the cell wall of *U. maydis* strains, wild type (*a2b2 wt*), mutant (*a2b2*∆*cda1*), and retro mutant (*a2b2*∆*cda1* + *CDA1*), was carried out by the method described by Ruiz-Herrera et al. [40] and Cifonelli [41].

### 2.6. Pathogenicity Tests

The experiments were made as previously described by Chavez-Ontiveros et al. [42]. Briefly, the experiments were conducted in a greenhouse using 7-day-old maize CV Cacahuazintle plants. *U. maydis* strains were grown under standard conditions in liquid complete medium supplemented with the appropriate selective agent (hygromycin B). Cells were recovered and washed by centrifugation with sterile double distilled water, and finally suspended in sterile distilled water. Sexually compatible strains, mixed in a 1:1 ratio (50 μL, 10^8^ cells/mL), were inoculated with a syringe and needle into the second node of the plant stem. Approximately 25 plants were inoculated with each mixture of the strains, and the experiments were repeated three times. Plants were incubated in a greenhouse under controlled conditions. Disease symptoms (chlorosis, anthocyanin formation, dwarf development, and presence of tumors) in the plants were scored after 14 days.

### 2.7. Microscopic Observations and Photographs

Cells were stained with one of the following solutions: calcofluor white or eosin, as described by Baker et al. [30], and were thoroughly washed by centrifugation, observed under UV light, and photographed with a DFC450C Leica camera.

### 2.8. Mating Assays

Mating was determined by the Fuz reaction [1] using plates of solid CM medium supplemented with 1% activated charcoal and sealed with parafilm. These were incubated at room temperature for 36 h, observed, and photographed.

### 2.9. Effect of Stress by Monovalent Cations on Cell Growth

To measure stress by monovalent cations, MM pH 7 plates containing 1.2 M LiCl, 1.0 M KCl, or 0.6M NaCl were inoculated with drops of serial decimal dilutions of cell suspensions containing 10^7^ to 10^3^ cells, as described above. Plates were incubated at 28 °C for 72 h and photographed.

### 2.10. Effect of Osmotic Stress on Cell Growth

The osmotic effect was analyzed using solid MM pH 7 plates with 1.5M sorbitol. Serial decimal dilutions of cell suspensions were inoculated as described above, incubated at 28 °C for 72 h, and photographed.

### 2.11. Dimorphism Induced by Acid pH

Induction of the dimorphic transition by a change in pH of growth was determined by the method described by Ruiz-Herrera et al. [43].

### 2.12. Phylogenetic Analysis

For phylogenetic analysis, fungal chitin deacetylase protein sequences were selected from the NCBI (https://www.ncbi.nlm.nih.gov/protein/ (accessed on 5 October 2021)) and JGI databases (https://jgi.doe.gov/our-science/science-programs/fungal-genomics/mycocosm-jgi-fungal-portal (accessed on 5 October 2021)). The name of each selected protein includes the name of the organism, followed by the protein identifier (ID), followed by the GPI in case of having the omega site; this is probably the glycosylphosphatidylinositol (GPI) anchor site.

The sequences obtained were aligned with the MAFFT program [44]. The MEGA 7 program [45] was used to obtain the dendrogram generated by the maximum likelihood method based on the Le_Gascuel model [46], with 500 bootstraps [47]. Phylogenetic dendrogram data were scaled with branch length in the same proportion as evolution distance. A discrete Gamma distribution was used to model the differences in evolutionary speed between sites (five categories (+G, parameter = 3.0476)). The rate variation model allowed some sites to be evolutionarily invariant ((+I), 0.79% sites) out of the 125 sequences. The phylogeny was rooted using the minimal deviation of ancestors (MAD) method [48]. The analyzed sequences and the tree in Newick format were deposited at https://github.com/lucilaortiz/CDAs_Ustilago (accessed on 23 April 2022).

## 3. Results

### 3.1. U. maydis CDAs Classification and Characterization

During our preliminary analyses of the transcriptome of the yeast-to-mycelium dimorphic transition of *U. maydis* at acid pH, we observed that among the genes encoding chitin deacetylases of the fungus, the gene encoding one of them (UM11922; CDA1) was highly up-regulated (fold change 40.4). Previously, it had been observed that the same gene was over-expressed during *A. thaliana* infection by *U. maydis* [32]. These results led us to consider the importance of chitin deacetylases in the development and pathogenicity of *U. maydis*, and to analyze the characteristics of these enzymes in the Basidiomycota in general.

**Figure 1:**
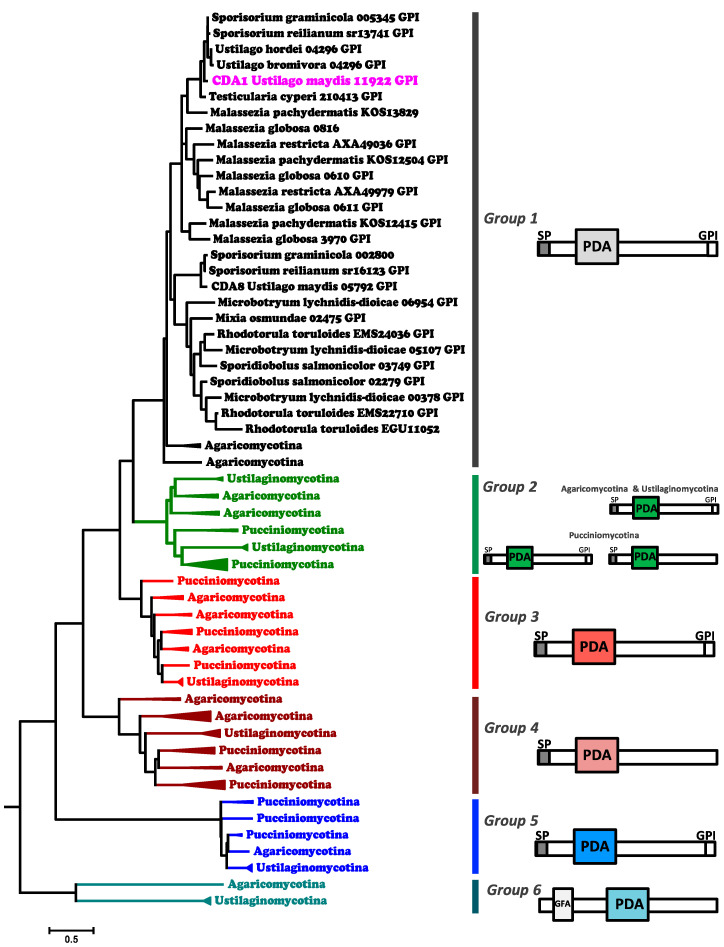
Compressed tree of Cda protein sequences from different Basidiomycota phyla. Based on 125 Cda sequences, the alignment was performed with MAFFT. The phylogenetic tree was reconstructed and compressed in MEGA7 and rooted with MAD. Most clades are collapsed, with the exception of group1, where Cda1 is present. The uncompressed tree is available in Supplementary Figure S2. On the right are schematic representations showing the organization of the domains in the Cda protein sequences. *GFA*, glutathione-dependent formaldehyde-activating; *GPI*, glycosil-phosphatidylinositol; *PDA*, polysaccharide deacetylase; *SP*, signal peptide.

In this work, we carried out the phylogenetic analysis of the chitin deacetylases (Cdas) of Basidiomycota. Our analysis included 125 protein sequences from twenty species of the subdivisions, Pucciniomycotina, Agaricomycotina, and Ustilaginomycotina. Using bioinformatic tools, we identified structural characteristic in the sequences of proteins, including: the functional domains (pfam, NCBI domain, SMART), the signal peptide (signalP 5.0), GPI-anchor motif (Pred-GPI), transmembrane helixes (TMHMM), and the prediction of cellular localization (psort wolf). The size, the position of the polysaccharide deacetylase 1 (PDA), and nod B domain in the sequence proteins were also considered (see Supplementary Table S1). The Cdas phylogenetic tree showed two principal clades: the first one including group 6, and the second one from the 1 to 5 group(s) (see Figure 1 and Figure S2).

The GPI anchor motif and the signal peptide were the most important features in the Cdas structures. Group(s) 3 and 5 have a GPI anchor; however, this motif is absent in group(s) 4 and 6. In contrast, most of the Cdas in group 1 have GPI, except *Malassezia globosa* and *Rhodotorula toruloides*, and all Cdas from Agaricomycotina and Ustilaginomycotina in group 2 have a GPI anchor, whereas this is variable in Pucciniomycotina. The presence of the signal peptide was only constant in group 5, except for the Cda of *Microbotryum lychnidis-dioicae*. On the other hand, the transmembrane helices were present in some Cdas from groups 1 to 5, but absent in group 6. Interestingly, additional domains to PDA were identified in the Ustilaginomycotina of group 6, which possess, in their structure, a GFA domain related to formaldehyde degradation, and Cda of *Coprinopsis cinerea* (group 4), which has a cellulose-binding motif (CBM). The prediction of the locations of the Cdas revealed that they were mostly extracellular; although, some of them are putatively localized in the cytoplasm or plasma membrane. The largest Cdas were those from groups 5 and 6 (500–750 aa); followed by those from groups 1, 2, and 3 (410–550 aa); and the smallest ones were those of group 4 (250 and 350 aa). Regarding the PDA domain, it was longer Cdas from groups 4, 5, and 6, and the smallest one in the Cdas from groups 1, 2, and 3 (see Supplementary Table S1). The Cdas of *U. maydis* and Ustilaginomycotina are phylogenetically distributed in each of the six groups: Cda2 family in group 6, Cda7 family in group 5, Cda5 family in group 4, Cda6 family in group 3, Cda4 and Cda3 families in group 2, and Cda1 and Cda8 families in group 1. However, Cdas from Malasseziomycetes were found in group 1.

### 3.2. CDA1 Is Involved in Mycelial Growth of U. maydis at Acid pH

**Figure 2:**
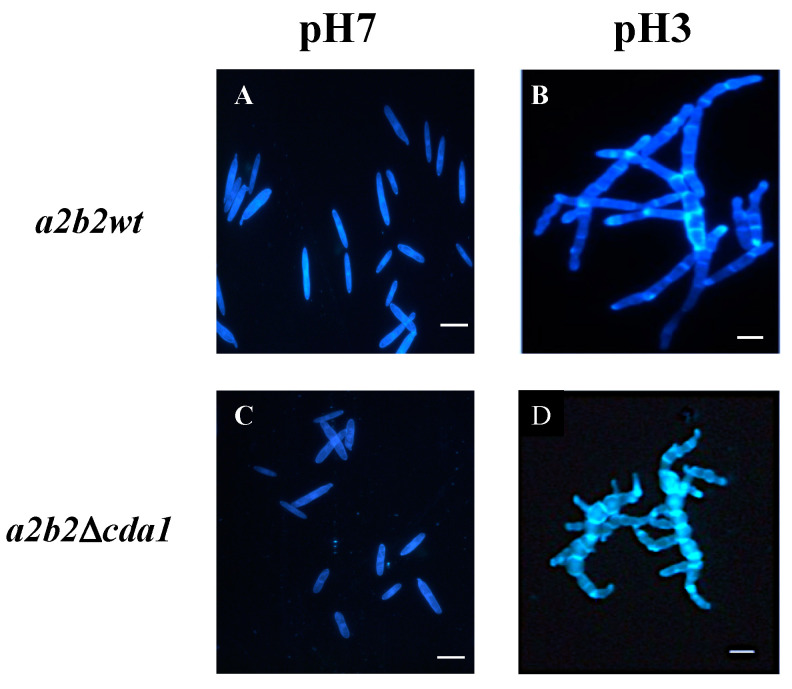
Cell morphology of *U. maydis* wild type and mutant strains grown in pH 7 or pH 3 minimal medium. (**A**) Wild type cells grown in pH 7 minimal medium and (**B**) grown in pH 3 medium; (**C**) cells of ∆*cda1* mutant grown al pH 7; (**D**) mutant grown at pH 3. All cells were stained with calcofluor white and photographed under UV light. Magnification bars, 10 μm.

During its life cycle, *U. maydis* presents two stages: one in the form of haploid saprophytic yeasts that divide by budding, and the other one that is the product of the mating of sexually compatible yeast cells (sporidia), in the form of mycelial dikaryons that invade the plant host. It was demonstrated that whereas the growth of the fungus at neutral or alkaline pH was in the yeast-like form, in acid pH medium, with a maximum of pH 3, *U. maydis* grows in the form of septate mycelium [43]. In this sense, *CDA1* mutation provoked abnormalities in the morphology of the mycelial growth of *U. maydis.* Thus, septa were considerably more abundant, and their morphology was bizarre. This result shows the important role of the deacetylase in the cell wall synthesis and organization (Figure 2). Surprisingly, in the yeast growth, no difference in structure was noticed between the wild type and the *cda1* mutant strain.

### 3.3. CDA1 Affects Chitosan Content and Provokes Chitosan Deposition in U. maydis Cells

**Figure 3:**
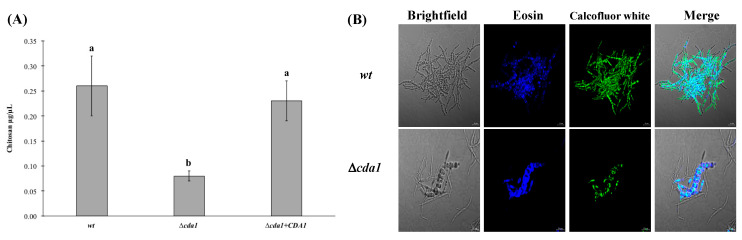
Determination of chitosan in the cell wall. (**A**) Chitosan quantification in *a2b2* wild type, ∆*cda1*, and ∆*cda1* + *CDA1* strains. (**B**) Cell morphology of *U. maydis* *a2b2 wt* and ∆*cda1* strains grown in pH 3 minimal medium. Cells were observed under brightfield, fluorescence with calcofluor white or eosin, and the merge. Results from four independent experiments with two replicas in each one. Bars represent standard error values. Different letters denote significant differences (ANOVA and Tukey’s test were performed, and reliability was 95%).

Cdas are metalloproteins that belong to an extracellular chitin-modifying enzyme family, carbohydrate esterase family 4 (CE-4), as shown in the carbohydrate active enzymes (CAZY) database, http://www.cazy.org (accessed on 22 November 2021) [49]. As mentioned above, the Cda1 enzymes deacetylate chitin, producing chitosan. The role of chitosan in the cell wall of the fungus is variable: it protects the spores of *Saccharomyces* against the attack of lytic enzymes [50], it provides the cell wall integrity of *Cryptococcus neoformans* [30], and it is involved in appressorium formation in *Magnaporthe oryzae* [29]. In order to determine the effect of *CDA*1 mutation on the content of chitosan, a quantification was carried out. It was observed that the amount of chitosan in the cell walls of the mutant strains (*a2b2*∆*cda1*) showed a decrease of 66.6% with respect to the amount of chitosan present in the *U. maydis* wild type cell walls (*a2b2wt*). The chitosan levels were recovered in the retro-mutant strain (*a*2*b*2∆*cda*1 + *CDA1*) to about 85% (Figure 3A). To observe the distribution of chitosan in the fungal cells, and the effect of the mutation of the deacetylase, wild type and mutant strains were stained with eosin. It was found that in the wild type (*a2b2wt*) strain grown at pH 3 and stained with eosin, the observed chitosan deposition was uniform throughout the entire hyphal body, whereas in the mutant strain (*a2b2*∆*cda1*), the distribution of chitosan was dispersed and unevenly distributed throughout the body of the aberrantly growing hyphae (Figure 3B).

### 3.4. CDA1 Is Involved in U. maydis Pathogenesis

**Figure 4:**
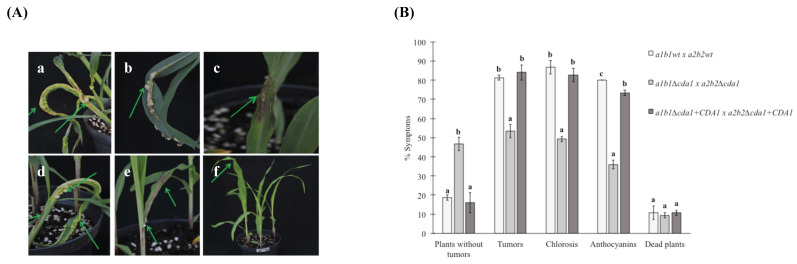
Virulence of Δ*cda1* mutant strain. (**A**) Macroscopic symptoms of plants. (**a**) Morphology of tumors induced in corn plants by the mixture of wild type strains *a1b1* × *a2b2*. (**b**,**c**) Disease symptoms induced by inoculation of the sexually compatible mixtures: *a1b1wt* × *a2b2*Δ*cda1*; (**d**) *a1b1*Δ*cda1* × *a2b2wt;* and (**e**,**f**) Δ*cda1* mutants *a1b1* × *a2b2*. (**B**) For each strain combination, three independent infection experiments were performed in the total of 75 infected plants. Symptoms were scored 14 days after infection (see Methods for details). The color code for disease rating is given at the bottom. Numbers at the right of the bars indicate the average percentage in each disease category. Arrows indicate areas of tumor. Bars represent standard error values. Different letters denote significant differences (ANOVA and Tukey´s test were performed, and reliability was 95%).

The greenhouse-grown corn seedlings inoculated with the sexually complementary mixture of wild type strains (*a1b1wt* × *a2b2wt*) of the *U. maydis* fungus formed large tumors, and induced chlorosis and anthocyanins. In some cases, the response to infection was so aggressive that the plants were killed (Figure 4A). In the case of the plants inoculated with the chitin deacetylase mutants (*a1b1*∆*cda1* × *a2b2*∆*cda1*), it was observed that the formation of tumors and the chlorosis and anthocyanin synthesis were decreased to about 50% with respect to the wild strains. The infection of plants with the sexually complementary mix of the chitin deacetylase mutant and wild type strain (*a1b1*∆*cda1* + *CDA1* × *a2b2*∆*cda1* + *CDA1*) showed that virulence and the other common features of disease were recovered at the same levels observed in the wild type strains (Figure 4B).

## 4. Discussion

Previously, we described that of the eight *CDA* genes present in the genome of *U. maydis*, only one of them (*CDA1*, UMAG_11922) was induced (40.4-fold change) in the mycelial form obtained by incubation of the fungus in acid medium [51] (Robledo-Briones and Ruiz-Herrera, 2013); the same occurred during *U. maydis* infection of *A. thaliana* seedlings, with fold changes from 10.9 to 31.0 [32]. Considering these results, we proceeded to analyze the importance of chitosan in the cell wall of *U. maydis* during the vegetative and infectious development of the fungus through the functional characterization of null mutants in the *CDA1* genes.

The phylogeny data of the Cdas let us hypothesize the evolutionary history of the Cdas families in Ustilaginomycetes. The Cdas of the Cda1, Cda3, Cda4, Cda6, and Cda8 families showed high similarity, all having a GPI anchor and a PDA domain. The data obtained suggest that Cdas from families Cda3 and Cda8 (arrow C and D, Figure S2) arose of the duplication of genes that encode Cda4 and Cda1, respectively, in a common ancestor of the species, *U. maydis*, *Sporisorium reilianum*, and *Sporisorium graminicola*, since there are no orthologs of these two families in other Ustilaginomycetes included in this study. Similarly, the data described in this work suggest that Cda1 and Cda4 families arose from the duplication of the gene encoding Cda6 (arrow A, Figure S2). The Cda4 family was putatively originated from a common ancestor before the appearance of *Testicularia cyperi* (arrow B, Figure S2); there is no ortholog of this family in that species, and the Cda1 family was putatively originated from a common ancestor of Ustilaginomycetes (see Supplementary Table S1).

The Cdas of family 7 have a larger size of the PDA domain (276 aa) and protein (550 aa). However, similar to the Cdas families described above, they have the GPI anchor motif, which suggests that they are evolutionarily related. In contrast, the Cdas of the Cda2 and Cda5 families showed greater differences; both lack a GPI anchor. Furthermore, Cda5 family members have a reduced size (300 aa) compared to other Cdas families from Ustilaginomycetes. These last three families (Cda2, Cda5, and Cda7) showed the highest conservation among their orthologs compared to the rest of the Cda families.

Analysis of the phenotype of the ∆*cda*1 mutants revealed that they developed an aberrant mycelium in pH acid media, and showed a greater number of branches compared to the wild type strain. Similarly, it was noticeable that both the content and the deposition of chitosan in the mycelium were severely altered. These results demonstrate the importance of chitosan for the correct formation of the mycelial wall structure of *U. maydis*. Likewise, it was observed that *CDA1* is responsible for the synthesis of a large part of the chitosan of the hyphae, since in its absence, its content decreased drastically (around 66.6%). Similarly to *U. maydis*, in *Magnaporthe oryzae*, it was observed that only the *CDA1* gene (of the ten putative genes present in its genome) was expressed at high levels in the vegetative mycelium [52]. However, contrary to *U. maydis* ∆*cda*1 mutants, the *M. oryzae* ∆*cda1* strains did not show morphological alterations in the mycelium. Notwithstanding this, the decrease in the content of chitosan in the cell wall was noticeable, since the staining of this polysaccharide was almost absent compared to the wild type strain, where chitosan strongly stained the septa and the lateral walls of the hyphae [52].

*U. maydis* ∆*cda*1 mutants also showed alterations in the virulence of the fungus, with a reduction of around 50%. Virulence was fully restored by re-insertion of the wild type *CDA1* gene into the ∆*cda1* mutants. These results show that *CDA1* is necessary for the complete virulence of *U. maydis*, probably by the reduction of the detection by its host. This result is similar to the phenomenon occurring in *Verticillium dahlia* and *Fusarium oxisporum*, where it was described that chitosan acted as a ¨sneaky molecule¨ that prevents the activation of the immune response during root infection [53]. In the phytopathogenic fungi, *Puccinia graminis*, *Uromyces fabae*, and *Colletotrichum graminicola*, it was also described that the modification of the cell wall surface by the deacetylation of chitin was a strategy to protect hyphae from hydrolysis by the chitinases produced by their hosts, as well as to avoid the defense response [54].

Unlike mycelium formation and the infection of *U. maydis* in maize seedlings, the growth of the ∆*cda*1 mutants was not affected by the presence of high concentrations of monovalent ions (Na^+^, K^+^, and Li^+^), nor by SDS or sorbitol. Moreover, the Fuz reaction was not affected in the mutants (Supplementary Figures S3 and S4, respectively). These results indicate that *CDA1* is not important for the response to stress by these compounds and conditions. Similar results were previously observed when studying this same *CDA1* gene (designated as *CDA3*), whose mutation did not induce susceptibility to: calcofluor, congo red, NaCl, sorbitol, caffeine, or SDS. However, unlike our data, the authors observed changes in virulence in *cda7* mutants. It should be noted that this is a mutant in the solopathogenic strain (SG200), and the Crisper-Cas9 method was used to obtain the mutant [55]; in our case, it was a knockout.

It is important to note that the deficiency of *CDA1* dramatically alters the morphology and pathogenesis of *U. maydis*, since microscopic observations on differential staining (eosin) of chitosan deposition showed that is aberrantly localized when grown at acid pH. In studies with the pathogen, *Cryptococcus neoformans*, it was observed that chitosan is distributed homogenously in the cell wall, whereas in the mutant that lacks three of the four genes of the same gene family, it shows altered behaviors in processes such as: separation in the cell wall, chitosan deposition in the cell wall, and pathogenesis in mice [56,57]. It is noteworthy that the simple mutant of *CDA1* of *U. maydis* shows a behavior similar to that of the triple mutant of *C. neoformans*; thus, we suggest that this gene is important in the differentiation process of the fungus from the formation of filamentous structures to the infection process. It is not the first time that a single gene belonging to a gene family is dominant in a particular cellular process. In the case of the chitin synthetase gene family, a similar phenomenon was found, when it was observed that chitin synthetase 6 showed a dominant role in virulence compared to the other seven members of the same gene family [17,58,59,60,61]. Moreover, it can be expected that genes belonging to gene families can partially recover the function of a gene that has a preponderant role in a specific process.

## 5. Conclusions

In conclusion, our results demonstrate that chitosan is an important component for the correct formation of the cell wall structure of the mycelial morphology, and to avoid recognition by its host during the pathogenic process of *U. maydis*.

## Data Availability

All relevant data are within the article and the Supplementary Material.

## Supplementary Materials

The following supporting information can be downloaded at: https://www.mdpi.com/article/10.3390/jof8080813/s1, Figure S1: Isolation of Δ*cda1* deletion construction and confirmation by PCR of *CDA1* mutation in the transformats; Figure S2: Phylogenetic tree of *Cda1* protein sequences from different Basidiomycota phyla; Figure S3: FUZ reaction between sexually compatible mixtures of wild type strains and the Δ*cda1* mutants; Figure S4: Effect of different types of stress; Table S1: Primers used in this study; Table S2: Domains and motifs of the Chitin deacetylases of Basidiomycota fungi.
