# Supplementary material for: Chitosan Is Necessary for the Structure of the Cell Wall, and Full Virulence of Ustilago maydis
Source: J Fungi (Basel). 2022 Aug 2;8(8):813. doi: 10.3390/jof8080813 (PMC9409902; doi:10.3390/jof8080813)
Supplement: Supplementary file 1 [file jof-08-00813-s001.zip › JoF Supplementary Figure S2. Phylogenetic tree of CDA protein.pdf]

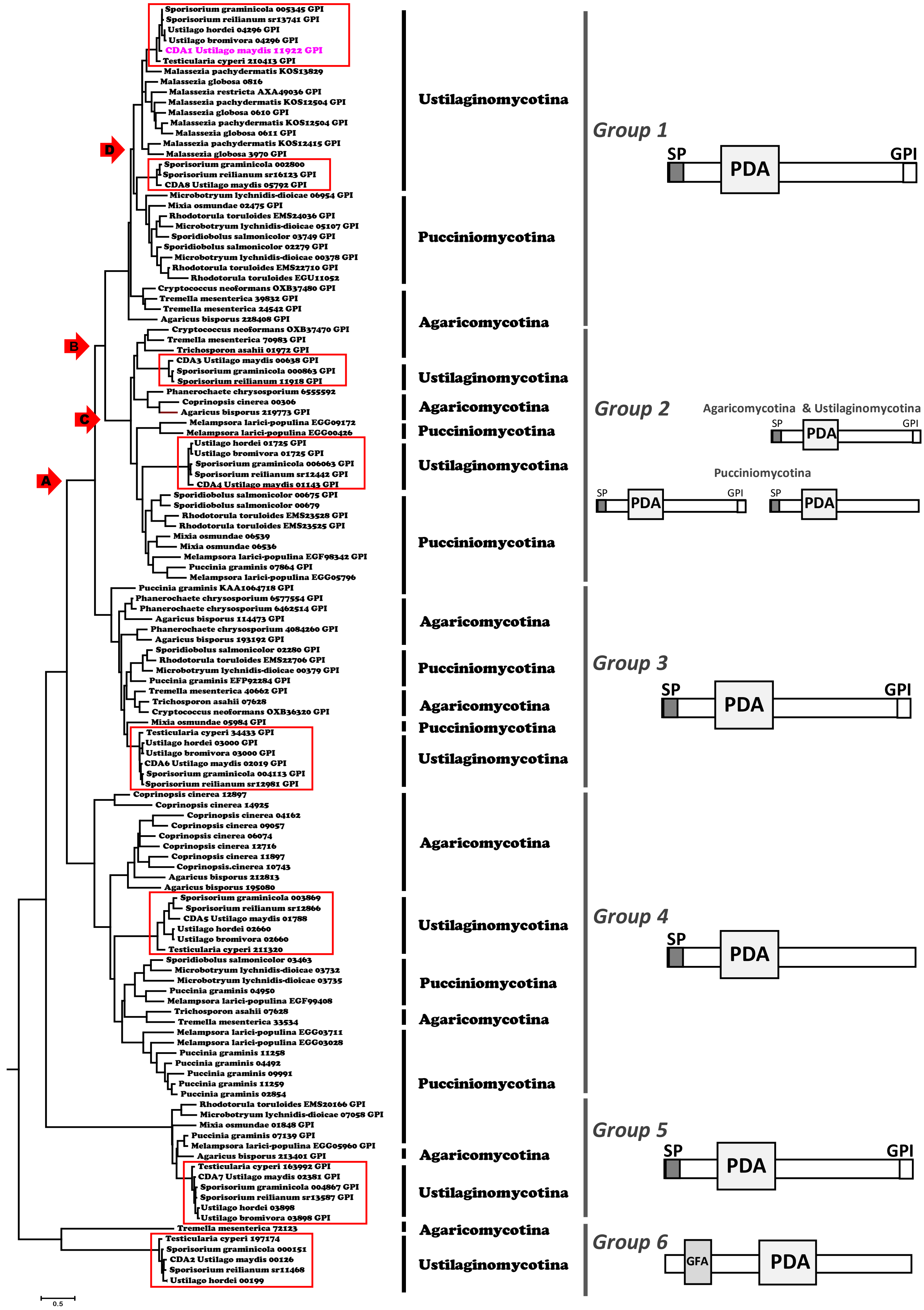

Sporisorium graminicola 005345 GPI
Sporisorium reilianum sr13741 GPI
Ustilago hordei 04296 GPI
Ustilago bromivora 04296 GPI
CDA1 Ustilago maydis 11922 GPI
Testicularia cyperi 210413 GPI
Malassezia pachydermatis KOS13829
Malassezia globosa 0816
Malassezia restricta AXA49036 GPI
Malassezia pachydermatis KOS12504 GPI
Malassezia globosa 0610 GPI
Malassezia pachydermatis KOS12504 GPI
Malassezia globosa 0611 GPI
Malassezia pachydermatis KOS12415 GPI
Malassezia globosa 3970 GPI
Sporisorium graminicola 002800
Sporisorium reilianum sr16123 GPI
CDA8 Ustilago maydis 05792 GPI
Microbotryum lychnidis-dioicae 06954 GPI
Mixia osmundae 02475 GPI
Rhodotorula toruloides EMS24036 GPI
Microbotryum lychnidis-dioicae 05107 GPI
Sporidiobolus salmonicolor 03749 GPI
Sporidiobolus salmonicolor 02279 GPI
Microbotryum lychnidis-dioicae 00378 GPI
Rhodotorula toruloides EMS22710 GPI
Rhodotorula toruloides EGU11052
Cryptococcus neoformans OXB37480 GPI
Tremella mesenterica 39832 GPI
Tremella mesenterica 24542 GPI
Agaricus bisporus 228408 GPI
Cryptococcus neoformans OXB37470 GPI
Tremella mesenterica 70983 GPI
Trichosporon asahii 01972 GPI
CDA3 Ustilago maydis 00638 GPI
Sporisorium graminicola 000863 GPI
Sporisorium reilianum 11918 GPI
Phanerochaete chrysosporium 6555592
Coprinopsis cinerea 00306
Agaricus bisporus 219773 GPI
Melampsora larici-populina EGG09172
Melampsora larici-populina EGG00426
Ustilago hordei 01725 GPI
Ustilago bromivora 01725 GPI
Sporisorium graminicola 006063 GPI
Sporisorium reilianum sr12442 GPI
CDA4 Ustilago maydis 01143 GPI
Sporidiobolus salmonicolor 00675 GPI
Sporidiobolus salmonicolor 00679
Rhodotorula toruloides EMS23528 GPI
Rhodotorula toruloides EMS23525 GPI
Mixia osmundae 06539
Mixia osmundae 06536
Melampsora larici-populina EGF98342 GPI
Puccinia graminis 07864 GPI
Melampsora larici-populina EGG05796
Puccinia graminis KAA1064718 GPI
Phanerochaete chrysosporium 6577554 GPI
Phanerochaete chrysosporium 6462514 GPI
Agaricus bisporus 114473 GPI
Phanerochaete chrysosporium 4084260 GPI
Agaricus bisporus 193192 GPI
Sporidiobolus salmonicolor 02280 GPI
Rhodotorula toruloides EMS22706 GPI
Microbotryum lychnidis-dioicae 00379 GPI
Puccinia graminis EFP92284 GPI
Tremella mesenterica 40662 GPI
Trichosporon asahii 07628
Cryptococcus neoformans OXB36320 GPI
Mixia osmundae 05984 GPI
Testicularia cyperi 34433 GPI
Ustilago hordei 03000 GPI
Ustilago bromivora 03000 GPI
CDA6 Ustilago maydis 02019 GPI
Sporisorium graminicola 004113 GPI
Sporisorium reilianum sr12981 GPI
Coprinopsis cinerea 12897
Coprinopsis cinerea 14925
Coprinopsis cinerea 04162
Coprinopsis cinerea 09057
Coprinopsis cinerea 06074
Coprinopsis cinerea 12716
Coprinopsis cinerea 11897
Coprinopsis.cinerea 10743
Agaricus bisporus 212813
Agaricus bisporus 195080
Sporisorium graminicola 003869
Sporisorium reilianum sr12866
CDA5 Ustilago maydis 01788
Ustilago hordei 02660
Ustilago bromivora 02660
Testicularia cyperi 211320
Sporidiobolus salmonicolor 03463
Microbotryum lychnidis-dioicae 03732
Microbotryum lychnidis-dioicae 03735
Puccinia graminis 04950
Melampsora larici-populina EGF99408
Trichosporon asahii 07628
Tremella mesenterica 33534
Melampsora larici-populina EGG03711
Melampsora larici-populina EGG03028
Puccinia graminis 11258
Puccinia graminis 04492
Puccinia graminis 09991
Puccinia graminis 11259
Puccinia graminis 02854
Rhodotorula toruloides EMS20166 GPI
Microbotryum lychnidis-dioicae 07058 GPI
Mixia osmundae 01848 GPI
Puccinia graminis 07139 GPI
Melampsora larici-populina EGG05960 GPI
Agaricus bisporus 213401 GPI
Testicularia cyperi 163992 GPI
CDA7 Ustilago maydis 02381 GPI
Sporisorium graminicola 004867 GPI
Sporisorium reilianum sr13587 GPI
Ustilago hordei 03898
Ustilago bromivora 03898 GPI
Tremella mesenterica 72123
Testicularia cyperi 197174
Sporisorium graminicola 000151
CDA2 Ustilago maydis 00126
Sporisorium reilianum sr11468
Ustilago hordei 00199
0.5
A
B
C
D
Ustilaginomycotina
Pucciniomycotina
Agaricomycotina
Ustilaginomycotina
Agaricomycotina
Pucciniomycotina
Ustilaginomycotina
Pucciniomycotina
Agaricomycotina
Pucciniomycotina
Agaricomycotina
Pucciniomycotina
Ustilaginomycotina
Agaricomycotina
Ustilaginomycotina
Pucciniomycotina
Agaricomycotina
Pucciniomycotina
Agaricomycotina
Ustilaginomycotina
Agaricomycotina
Ustilaginomycotina
Group 1
SP
PDA
GPI
Group 2
Agaricomycotina & Ustilaginomycotina
SP
PDA
GPI
Pucciniomycotina
SP
PDA
GPI
SP
PDA
Group 3
SP
PDA
GPI
Group 4
SP
PDA
Group 5
SP
PDA
GPI
Group 6
GFA
PDA
