# Supplementary material for: Chitosan Is Necessary for the Structure of the Cell Wall, and Full Virulence of Ustilago maydis
Source: J Fungi (Basel). 2022 Aug 2;8(8):813. doi: 10.3390/jof8080813 (PMC9409902; doi:10.3390/jof8080813)
Supplement: Supplementary file 1 [file jof-08-00813-s001.zip › JoF Supplementary Table S1 Primers.pdf]

Supplementary Table. S1. Primers used in this study

	ID		SEQUENCE
**A**	CDS1-PF1	Forward	CGACCAACGCCTCGGCACAG
**B**	CDS1-PQR2	Reverse	CGAACCCCACCTTCTTCCGCGATCTGATGAGTAAATGCTAGTGATGGTCG
**C**	CDS1-UQF3	Forward	CGTACGAAAGCGAGACGAGTTGAGCGCCTAGATCCCTTGTAATCT
**D**	CDS1-UTR4	Reverse	CGGTCGTCATCGCTTCTAA
**E**	pHyg101Hygromycin-F	Forward	CTCGAGTGGCGGCAGATGTGAGT
**F**	pHyg101Hygromycin-R	Reverse	GGTTCACTTACTCCACGTTG
**G**	CDS1-NF5	Forward	GCTGACTGTCTTGCTGGCG
**H**	CDS1-NR6	Reverse	CGAACAAGCCAGACACTGT
