# Supplementary material for: Chitosan Is Necessary for the Structure of the Cell Wall, and Full Virulence of Ustilago maydis
Source: J Fungi (Basel). 2022 Aug 2;8(8):813. doi: 10.3390/jof8080813 (PMC9409902; doi:10.3390/jof8080813)
Supplement: Supplementary file 1 [file jof-08-00813-s001.zip › JoF Supplementary Table S2 Domains and motifs Basidiomycota.pdf]

Supplementary Table S2. Domains and motifs of the Chitin deacetylases of Basidiomycota fungi.

Group	Subdivision	Fungus	ID	Protein size	SP	PDA			GPI	TMH	Others	Localization
						Start	End	Size				
1	U	*S. graminícola*	005345	434	Yes	178	361	184	Yes	-	-	EC
		S. relianum	sr13741	490	Yes	178	361	184	Yes	-	-	EC
		U. hordei	04296	490	Yes	178	361	184	Yes	-	-	EC
		U. bromivora	04296	490	Yes	178	361	184	Yes	-	-	EC
		U. maydis	**11922 (Cda1)**	**597**	**-**	**288**	**471**	**184**	**Yes**	**-**	**-**	**EC**
		T. cyperi	210413	487	Yes	175	358	184	Yes	-	-	EC
		M. pachydermatis	KOS13829	424	-	118	293	176	Yes	-	-	PM
		M. globosa	0816	424	Yes	173	351	179	-	-	-	EC
		M. restricta	AXA49036	493	Yes	156	329	174	Yes	-	-	EC
		M. pachydermatis	KOS12504	451	-	113	290	178	Yes	-	-	C
		M. globosa	0610	472	Yes	155	328	174	Yes	-	-	EC
		M. restricta	AXA49979	486	Yes	166	340	175	Yes	-	-	EC
		M. globosa	0611	465	Yes	162	336	175	Yes	-	-	EC
		M. pachydermatis	KOS12415	561	-	253	436	184	Yes	-	-	EC
		M. globosa	3970	457	Yes	162	344	166	Yes	Yes	-	C
		S. graminícola	002800	557	Yes	232	414	183	Yes	Yes	-	EC
		S. relianum	sr16123	528	-	204	386	183	Yes	Yes	-	PM
		U. maydis	05792 (Cda8)	462	-	159	324	166	Yes	-	-	PM
	P	*M. lychnidis-dioicae*	06954	561	Yes	135	309	175	Yes	-	-	EC
		M. osmundae	02475	514	-	181	361	182	Yes	-	-	EC
		R. turuloides	EMS24036	464	Yes	159	338	180	Yes	-	-	EC
		M. lychnidis-dioicae	05107	489	Yes	163	341	179	Yes	Yes	-	EC
		S. salmonicolor	03749	575	Yes	151	408	258	Yes	-	-	EC
		S. salmonicolor	02279	473	Yes	173	348	176	Yes	Yes	-	EC
		M. lychnidis-dioicae	00378	472	Yes	153	333	181	Yes	-	-	EC
		R. turuloides	EMS22710	482	Yes	182	361	180	Yes	Yes	-	EC
		R. turuloides	EGU11052	595	-	158	309	152	-	-	-	EC
	A	*C. neoformans*	OXB37480	462	Yes	158	339	182	Yes	-	-	EC
		T. mesentérica	39832	445	Yes	159	343	185	Yes	-	-	EC
		T. mesentérica	24542	448	Yes	159	340	182	Yes	-	-	EC
		A. bisporus	228408	426	Yes	128	309	182	Yes	Yes	-	EC
2	A	*C. neoformans*	OXB37470	412	Yes	124	307	184	Yes	-	-	EC
		T. mesentérica	70983	406	Yes	120	303	184	Yes	-	-	EC
		T. asahii	01972	429	Yes	129	305	177	Yes	-	-	EC
	U	*U. maydis*	00638 (Cda3)	425	Yes	110	298	189	Yes	-	-	EC
		S. graminícola	000863	420	Yes	108	296	189	Yes	-	-	EC
		S. relianum	11918	422	Yes	107	295	189	Yes	Yes	-	EC
	A	*P. chrysosporium*	6555592	493	Yes	153	335	183	Yes	Yes	-	EC
		C. cinerea	00306	406	Yes	153	326	174	-	-	-	EC
		A. bisporus	219773	436	Yes	143	321	178	Yes	Yes	-	EC
	P	*M. larici-populina*	EGG09172	410	-	181	385	205	-	-	-	EC
		M. larici-populina	EGG00426	433	-	217	407	191	-	Yes	-	C
	U	*U. hordei*	01725	485	Yes	139	331	193	Yes	-	-	EC
		U. bromivora	01725	481	Yes	139	331	193	Yes	-	-	EC
		S. graminícola	006063	472	Yes	140	333	194	Yes	-	-	EC
		S. relianum	12442	478	Yes	140	334	195	Yes	Yes	-	EC
		U. maydis	01143 (Cda4)	480	Yes	142	334	193	Yes	-	-	EC
	P	*S. salmonicolor*	00675	338	-	33	220	188	Yes	Yes	-	EC

		S. salmonicolor	00679	274	-	150	264	115	-	-	-	EC
		R. turuloides	EMS23528	409	Yes	100	287	188	Yes	-	-	EC
		R. turuloides	EMS23525	402	Yes	106	294	189	Yes	-	-	EC
		M. osmundae	06539	616	Yes	141	330	190	-	Yes (2)	-	EC
		M. osmundae	06536	434	-	87	262	176	-	Yes	-	C
		M. larici-populina	EGF98342	428	Yes	106	294	189	Yes	-	-	EC
		P. graminis	07864	432	Yes	117	318	202	Yes	-	-	EC
		M. larici-populina	EGG05796	330	Yes	120	313	194	-	-	-	C
**3**	**P**	*P. graminis*	KAA1064718	430	Yes	148	334	187	Yes	-	-	EC
	**A**	*P. chrysosporium*	6577554	479	Yes	197	383	187	Yes	-	-	EC
		P. chrysosporium	6462514	499	Yes	232	419	188	Yes	-	-	EC
		A. bisporus	114473	313	-	239	424	186	Yes	-	-	C
		P. chrysosporium	4084260	475	Yes	207	385	179	Yes	-	-	EC
		A. bisporus	193192	444	Yes	176	354	179	Yes	Yes	-	EC
	**P**	*S. salmonicolor*	02280	526	Yes	213	383	171	Yes	-	-	EC
		R. turuloides	EMS22706	498	Yes	193	380	188	Yes	-	-	EC
		M. lychinidias-dioicae	00379	490	Yes	188	370	183	Yes	-	-	EC
		P. graminis	EFP92284	471	-	200	380	181	Yes	-	-	EC
	**A**	*T. mesenterica*	40662	490	Yes	182	372	191	Yes	-	-	EC
		T. asahii	05842	472	Yes	183	369	187	Yes	-	-	EC
		C. neoformanas	OXB36320	470	Yes	159	350	192	Yes	Yes	-	EC
	**P**	*M. osmundae*	05984	524	Yes	254	432	179	Yes	-	-	EC
	**U**	*T. cyperi*	34433	474	Yes	191	370	180	Yes	-	-	EC
		U. hordei	03000	479	Yes	195	374	180	Yes	Yes	-	EC
		U. bromivora	03000	480	Yes	196	375	180	Yes	Yes	-	EC
		U. maydis	02019 (Cda6)	466	Yes	185	364	180	Yes	Yes	-	EC
		S. graminicola	004113	472	Yes	184	363	180	Yes	-	-	EC
		S. relianum	sr12981	474	Yes	185	364	180	Yes	Yes	-	EC
**4**	**A**	*C. cinerea*	12897	306	-	86	278	193	-	-	CBM	C
		C. cinerea	14925	265	Yes	43	235	193	-	-	-	EC
		C. cinerea	04162	398	Yes	46	297	252	-	Yes (2)	-	PM
		C. cinerea	09057	255	Yes	36	230	195	-	-	-	EC
		C. cinerea	06074	290	Yes	33	227	195	-	-	-	EC
		C. cinerea	12716	271	Yes	54	247	194	-	-	-	EC
		C. cinerea	12897	306	-	86	278	193	-	-	CBM	C
		C. cinerea	10743	262	Yes	42	232	191	-	-	-	EC
		A. bisporus	212813	260	-	43	230	188	-	-	-	C
		A. bisporus	195080	255	Yes	37	231	195	-	-	-	EC
	**U**	*S. graminicola*	003869	302	Yes	75	265	191	-	-	-	EC
		S. relianum	sr12866	302	Yes	75	265	191	-	-	-	EC
		U. maydis	01788 (Cda5)	304	Yes	76	266	191	-	-	-	EC
		U. hordei	02660	303	Yes	76	266	191	-	-	-	EC
		U. bromivora	02660	303	Yes	76	266	191	-	-	-	EC
		T. cyperi	211320	301	Yes	75	264	190	-	-	-	EC
	**P**	*S. salmonicolor*	03463	281	Yes	57	247	191	-	-	-	EC
		M. lychinidias-dioicae	03732	273	Yes	46	239	194	-	-	-	EC
		M. lychinidias-dioicae	03735	360	-	41	231	191	-	-	-	C
		P. graminis	04950	269	Yes	38	229	192	-	-	-	EC
		M. larici-populina	EGF99408	270	Yes	36	230	195	-	-	-	EC
	**A**	*T. asahii*	07628	246	Yes	29	219	191	-	-	-	EC
		T. mesenterica	33534	248	Yes	30	221	192	-	-	-	EC
	**P**	*M. larici-populina*	EGG03711	291	-	46	252	207	-	-	-	C
		M. larici-populina	EGG03028	424	Yes	194	383	190	-	-	-	EC

		P. graminis	11259	313	Yes	83	271	189	-	Yes	-	EC
		P. graminis	04492	287	Yes	57	248	192	-	-	-	EC
		P. graminis	09991	323	-	88	279	192	-	Yes	-	EC
		P. graminis	11258	289	Yes	59	250	192	-	-	-	EC
		P. graminis	02854	321	-	91	282	192	-	Yes	-	EC
**5**	**P**	*R. turuloides*	EMS20166	505	Yes	72	342	271	Yes	-	-	EC
		M. lychinidias-dioicae	07058	602	-	106	412	307	Yes	Yes	-	C
		M. osmundae	01848	758	Yes	75	346	272	Yes	-	-	EC
		P. graminis	07139	520	Yes	89	360	272	Yes	-	-	EC
		M. larici-populina	EGG05960	526	Yes	92	363	272	Yes	-	-	EC
	**A**	*A. bisporus*	213401	512	Yes	63	340	278	Yes	Yes	-	C
	**U**	*T. cyperi*	163992	561	Yes	106	381	276	Yes	Yes	-	EC
		U. maydis	02381 (Cda7)	548	Yes	94	369	276	Yes	Yes	-	EC
		S. graminicola	004867	550	Yes	96	371	276	Yes	Yes	-	EC
		S. relianum	sr13587	550	Yes	96	371	276	Yes	Yes	-	EC
		U. hordei	03898	548	Yes	94	369	276	Yes	Yes	-	EC
		U. bromivora	03898	548	Yes	94	368	276	Yes	Yes	-	EC
**6**	**A**	*T. mesenterica*	72123	342	-	26	311	288	-	-	-	C
	**U**	*T. cyperi*	197174	526	-	152	379	228	-	-	GFA	C
		S. graminicola	000151	525	-	150	378	229	-	-	GFA	C
		U. maydis	00126 (Cda2)	525	-	149	377	229	-	-	GFA	C
		S. relianum	sr11468	525	-	151	379	229	-	-	GFA	C
		U. hordei	00199	523	-	150	378	229	-	-	GFA	C
		U. bromivora	00199	522	-	150	378	229	-	-	GFA	C

SP: signal peptide, PDA: polysaccharide deacetylase 1, GPI: GPI-anchor, TMH: transmembrane helix, GFA: Glutathione-dependent formaldehyde-activating enzyme, CBM: Cellulose binding domain; A: Agaricomycotina, P: Pucciniomycotina, U: Ustilaginomycotina; EC: extracellular, C: cytoplasm, PM: plasma membrane.
