# Supplementary material for: Chitosan Is Necessary for the Structure of the Cell Wall, and Full Virulence of Ustilago maydis
Source: J Fungi (Basel). 2022 Aug 2;8(8):813. doi: 10.3390/jof8080813 (PMC9409902; doi:10.3390/jof8080813)
Supplement: Supplementary file 1 [file jof-08-00813-s001.zip › Supplementary Figure Captions.pdf]

Figure S1. Obtention of ⊿*cda*1 deletion construction and confirmation by PCR of *CDA1* mutation in the transformants. **(A)** Amplification of 5′(1.2 Kb) and 3′(1.2 Kb) regions of *CDA1* gene and HPH marker (2.3 Kb) in the first PCR reaction; **(B)** ⊿*cda1* deletion construction (4.5 Kb). **(C)** Amplification with nested primers G and H (Supplementary Table S1) Line1, DNA of wild type strain (3.7 Kb) was used as negative control; line 7, as positive control the ⊿*cda1* deletion construction was used; lines 2-6, amplification in the transformants. **(D)** Amplification of 150 pb out of the construction in the 5′flanking region using the A and F primers (Supplementary Table S1). The size of the expected bands is 3.6 Kb; as a negative control we used DNA of the wild strain (line 1); lines 2-6, amplification in the transformants. The strains ⊿*cda1* of the lines 3-6 were selected for the study of *CDA1* gene.

Figure S2. Phylogenetic tree of *Cda1* protein sequences from different Basidiomycota phyla. (See details in the Figure 1). The arrows indicated the branching points for duplications (see discussion) and the identification numbers was described in materials and methods. Ustilaginomycetes are highlighted within the squares (represented a families). On the right are schematic representation showing the organization of the domains in the Cda protein sequences. *GFA* glutathione-dependent formaldehyde-activating, *GPI* glycosilphosphatidylinositol, *PDA* polysaccharide deacetylase, *SP* signal peptide.

Figure S3. FUZ reaction between sexually compatible mixtures of wild type strains and the ⊿*cda1* mutants.

Figure S4. Effect of different types of stress. Decimal dilutions of cell suspensions (107) cells/mL) (*a2b2*) wild type strain and (*a2b2*) ⊿*cda1* mutant were spotted on MM pH7 plates and grown for 72 h at 28℃. 1.5 M Sorbitol; 5mM LiCl; 1M NaCl.

Table S1. Primers used in this study.

Table S2. Domains and motifs of the Chitin deacetylases of Basidiomycota fungi.
