# Supplementary figures and images for: Chitosan Is Necessary for the Structure of the Cell Wall, and Full Virulence of Ustilago maydis
Source: J Fungi (Basel). 2022 Aug 2;8(8):813. doi: 10.3390/jof8080813 (PMC9409902; doi:10.3390/jof8080813)

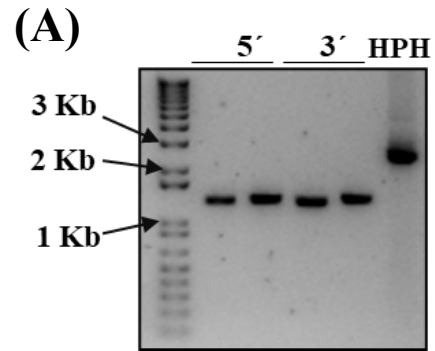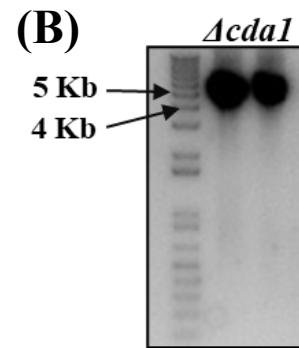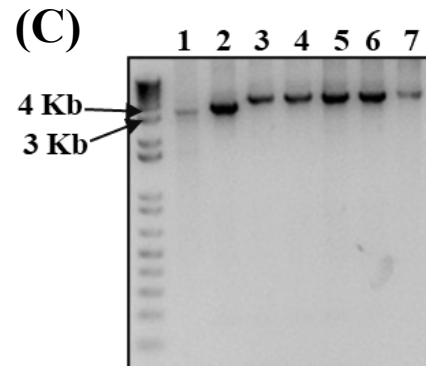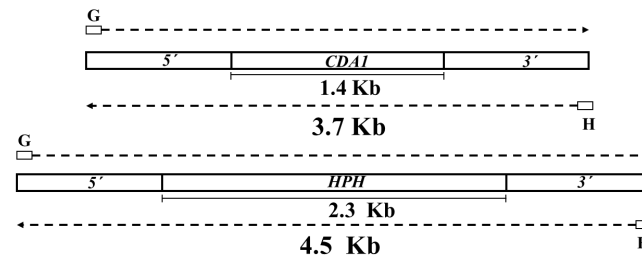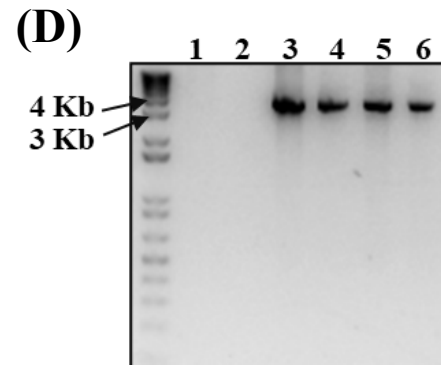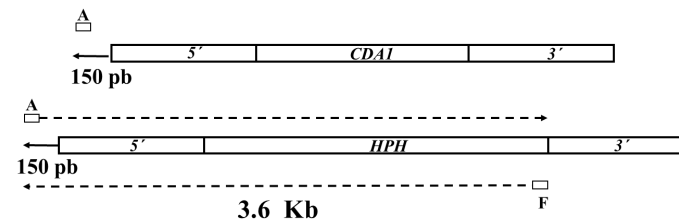

Supplement: Supplementary file 1 [file jof-08-00813-s001.zip › JoF Supplementary Figure S1. Obtention of ...cda1 deletion construction.pdf]

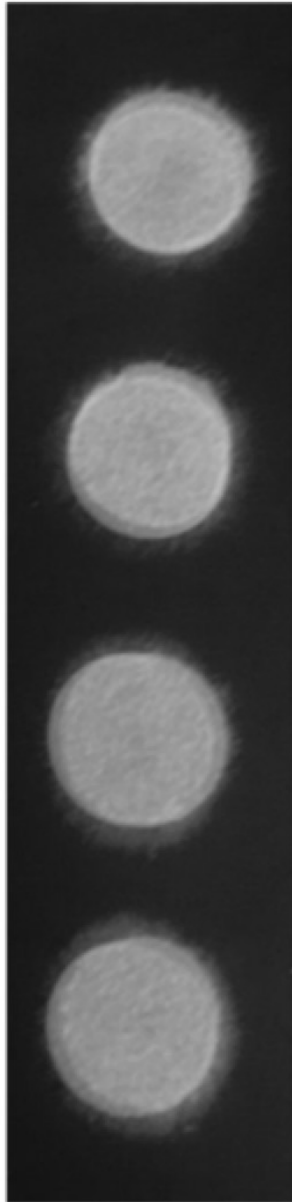

*a1b1wt* x *a2b2wt*

*a1b1wt* x *a2b2Δcda1*

*a1b1Δcda1* x *a2b2wt*

*a1b1Δcda1* x *a2b2Δcda1*

Supplement: Supplementary file 1 [file jof-08-00813-s001.zip › JoF Supplementary Figure S3 Fuz.pdf]

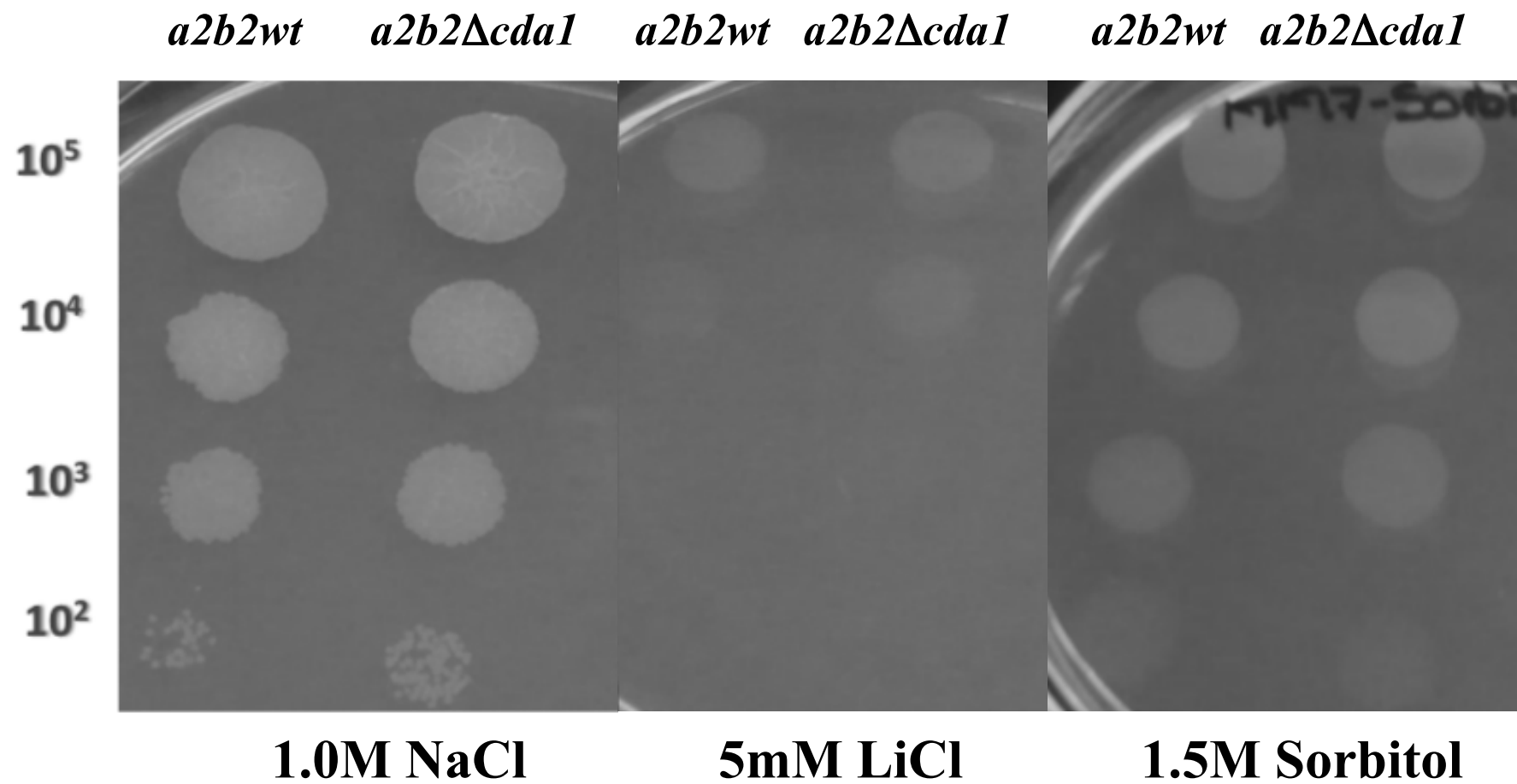

Supplement: Supplementary file 1 [file jof-08-00813-s001.zip › JoF Supplementary Figure S4 Stresses.pdf]
